# Attitudes of medical students towards choosing psychiatry as a career

**DOI:** 10.1192/bjo.2021.70

**Published:** 2021-06-18

**Authors:** Mohammad Ahmad, Marwan Dabbagh, Alawwab Dabaliz, Akef Obeidat

**Affiliations:** 1 Barnet Enfield and Haringey MH NHS Trust, Lancashire Care NHS Foundation Trust; 2 Alfaisal University; 3 University Hospitals, Cleveland Medical Center

## Abstract

**Aims:**

Our aim is to study factors influencing attitudes of medical students towards pursuing Psychiatry as a career. We hypothesise that the minimal exposure and/or importance given to Psychiatry during medical school is insufficient to let a student truly experience and appreciate the specialty.

**Background:**

Studies report an annual decline in Psychiatry Trainees in many parts of the world. This deficiency is projected to create gaps between mental health service needs and providers. Studies have also explored the crisis in recruitment and the positive impact a short course can have in promoting engagement in Psychiatry by students.

**Method:**

An anonymous questionnaire was distributed amongst medical students, from years 1 to 5, in the College of Medicine, Alfaisal University, Riyadh, Saudi Arabia. Factors assessed in the survey included demographics, specialty ranking, acceptance ratios, role models and personal experiences, among others. Those who didn't express interest were asked about the lack of exposure to Psychiatry during medical school along with other influential factors that have been studied elsewhere, including those that we hypothesise to be of significance in our study population.

**Result:**

A total of 153 students responded. Positive views towards Psychiatry increased linearly by year (50% in Year 1 to 90% in Year 5). 33% of students selected psychiatry as a top 3 choice with the most significant factors being a unique patient-doctor relationship (P < 0.05), and the challenges faced in the specialty (95%).

Of the 67% of students who did not prefer Psychiatry, insufficient exposure to ward experiences and the specialty as a whole were unanimously agreed upon factors. Other deterring factors included lack of instant gratification when treating a psychiatric patient (72%), and an underestimation by the non-medical community of a Psychiatrists role (26%).

**Conclusion:**

Our findings give an optimistic view towards the future of Psychiatry in the region, given the large number of students (33%) who consider it in their top 3 choices for a career. However, a larger number of students continue to have a negative view towards Psychiatry, especially due to the lack of exposure to the specialty during medical school. The factors identified in our study should be tackled by medical schools or curriculum provision authorities, as this has shown to be of benefit in studies in other parts of the world.

